# Sexual dimorphism in the osmopressor response following water ingestion

**DOI:** 10.1042/BSR20150276

**Published:** 2016-06-17

**Authors:** Goncalo V. Mendonca, Carolina Teodósio, Rui Lucena, Fernando D. Pereira

**Affiliations:** * CIPER: Laboratory of Motor Behavior, Faculdade de Motricidade Humana, Universidade de Lisboa, Lisboa, 1499-002 Cruz-Quebrada Dafundo, Portugal; † Laboratory of Motor Behavior, Faculdade de Motricidade Humana, Universidade de Lisboa, Lisboa, 1499-002 Cruz-Quebrada Dafundo, Portugal; ‡ Centro de investigação da Academia Militar (CINAMIL), 1169-203 Lisboa, Portugal; § CIPER, Faculdade de Motricidade Humana, Universidade de Lisboa, Lisboa, 1499-002 Cruz-Quebrada Dafundo, Portugal

**Keywords:** cardiovascular function, heart rate variability, haemodynamics

## Abstract

There is conflicting evidence as to whether water drinking elicits a pressor response in healthy young adults. The inclusion of a variable number of women may have contributed to the discrepancies found in past research. Thus, we aimed at exploring whether the osmopressor response follows a sexually dimorphic pattern. In a randomized fashion, 31 healthy adults (16 men; 15 women, aged 18–40 years) ingested 50 and 500 ml of water before completing a resting protocol on two separate days. Arterial blood pressure, heart rate and spectral heart rate variability were measured in the seated position at pre- and post-25 min of water ingestion. Women responded to 500 ml of water with a greater proportion of change in diastolic and mean arterial pressure (MAP) (*P*<0.05). Conversely, the percent change in systolic blood pressure (SBP) and heart rate was not different between sexes after 500 ml of water. Overall, women demonstrated lower blood pressure, but higher resting heart rate compared with men (*P*<0.05). In contrast, heart rate variability was similar between sexes before and after ingesting either volume of water. There was a bradycardic effect of water and, irrespectively of sex; this was accompanied by increased high frequency power (HF) (*P*<0.05). We conclude that women display a greater magnitude of pressor response than men post-water ingestion. Accordingly, we provide direct evidence of sexual dimorphism in the haemodynamic response to water intake in young healthy adults.

## Abstract (precis)

Compared with men, women exhibit a greater magnitude of increase in resting blood pressure after drinking a single water bolus of 500 ml. Accordingly, our study provides direct evidence of sexual dimorphism in the haemodynamic response to water intake.

## INTRODUCTION

There is general agreement that water ingestion causes many physiological changes in the human body, including fluctuations in endocrine function, cardiovascular autonomic regulation and fluid balance [1–4]. For example, drinking ∼500 ml of water augments sympathetic outflow, as shown by a robust increase in muscle sympathetic nerve activity (MSNA), calf vascular resistance, plasma noradrenaline (norepinephrine) and salivary α-amylase activity [2,4–6]. It has been previously reported that the haemodynamic effects of drinking 500 ml of water are largely attenuated in liver transplant recipients, but remain well preserved in in patients with high-level spinal cord injuries [7,8]. This indicates that the vascular response to water ingestion is triggered by hypoosmolarity within the hepatic portal system and may involve a spinal reflex-like mechanism [7–10]. As importantly, recent research has also shown that the autonomic response to water ingestion is enhanced by drinking cold- and room-tempered water, but not body-tempered water [11].

Jordan et al. [12] were the first to note that, in persons with autonomic failure, the severity of orthostatic intolerance was substantially decreased shortly after water ingestion. Hypertensive patients have also been shown to respond to water ingestion with an acute rise in blood pressure (BP) [13]. A more discrete, but nonetheless significant, pressor effect was additionally described for middle-aged and elderly persons [2]. Conversely, conflicting data have been reported on the haemodynamic response to water ingestion in young healthy individuals. Although some authors observed no changes in BP post-water ingestion [3,4], others reported a small, but significant pressor effect at resting conditions [5,6,11,13,14]. Importantly, none of these studies explored the association between sexual dimorphism and the increase in resting BP secondary to water ingestion.

There is compelling evidence that both sexes exhibit considerable differences in autonomic regulation and that the menstrual cycle affects peripheral vasoconstriction [15–19]. Thus, the inclusion of a variable number of women and the lack of control for the effects of menstrual cycle on the haemodynamic response to water ingestion may have contributed to the discrepancies found in past research. Remarkably, the hypothetical interaction between sex and the physiological response to water drinking is strongly supported by one previous investigation examining the impact of water ingestion on resting metabolism [20]. For this reason, we tested the hypothesis that drinking a single water bolus (500 ml of mineral water) elicits a greater magnitude of pressor response in healthy young women compared with that seen in men.

## MATERIALS AND METHODS

### Participants

Thirty-one healthy individuals, aged 18–40 years (16 men and 15 women), participated in the present study. All participants were nonsmokers and normotensive (systolic and diastolic BP values repeatedly <135/85 mmHg) [21]. Participants were all non-obese and free of any known cardiovascular or metabolic disease, as assessed by medical history. None of the participants were currently using prescription or taking any medications. All women were tested during the early follicular phase of the menstrual cycle (days 2–6) to control for possible effects of menstrual cycle on vasoreactivity and heart rate variability. None of them were pregnant or using oral contraceptives at the time of the study. Additionally, they all had self-reported regular menstrual cycles of ∼28 days. Each participant was requested to avoid heavy exercise for at least 24 h before testing and to have nothing to drink or eat from midnight until the testing session on the subsequent morning. Additionally, participants were asked to consume 1 litre of water ∼8 h before each visit (just before midnight) to ensure adequate hydration [22]. Written informed consent was obtained before study entry. The present experimental design was carried out in accordance with the Declaration of Helsinki and was approved by the University's Institutional Review Board.

### Study design

Participants were evaluated over the course of two visits, on separate days, at approximately the same time of the day (between 07.00 and 11.00 h). Participants acted as their own controls and, in a randomized counterbalanced manner, they were all tested at resting conditions on two different occasions within a 72-h period: (1) post-ingestion of 50 ml of water and (2) post-ingestion of 500 ml of water. On each visit, after arriving to the laboratory, participants were asked to empty their bladders. This was done with the purpose of avoiding urinary bladder distension, which is known to affect peripheral sympathetic activity. During the first visit, standing height and body mass measurements were taken with the participants wearing light-weight clothes and no shoes. Height was taken using a stadiometer with measures obtained to the nearest 0.5 cm. Body mass was measured on a digital scale to the nearest 0.01 kg (BG 42, Breuer GmbH). Body mass index (BMI) was then calculated by dividing the participants’ mass in kilograms by the square of their height in meters.

There is general agreement that the osmopressor response appears within 5–10 min, is maximal at 25–40 min, and largely dissipates by 90 min of water drinking [2]. For this reason, the 25-min time point post-water ingestion was taken as representative of the peak osmopressor response in comparison with baseline level before water ingestion. Specifically, after 15-min of rest, testing was started with a 10-min resting period in the seated position (baseline). Subsequently, participants ingested either 50 (control) or 500 ml (experimental) of mineral water (Água Mineral de Luso) at room temperature within 90 s and remained quietly seated for another 25 min. None of the participants reported feeling any discomfort related to the ingestion of either volume of water in such a short period of time. Testing was carried out in the laboratory with an environmental temperature between 21 and 24°C and a relative humidity between 44 and 56%.

### Measurements

R–R intervals and brachial BP were obtained during the last 5 and 3 min of each resting period (pre- and post-water ingestion) respectively. Past research indicates that spectral heart rate variability is affected by minute ventilation [23]. For this reason, during these measurements, participants’ breathing frequency was paced at 12 breaths per minute with the aid of a metronome. R–R interval data were recorded by means of a Polar RS 800 G3 heart rate monitor (Polar R–R Recorder, Polar Electro) and resting values of systolic and diastolic BP were measured with an automated BP monitor, in duplicate (Tango SunTech, Medical). For analysis, the average of the two resting BP values was used. If the values were not within 5 mmHg, a third measurement was taken and the two closest values were averaged and used for analysis. All measurements were performed with each participant seated comfortably, with back supported, legs uncrossed and upper arm bare. As recommended by Pickering et al. [21], cuff size was selected based on the participants’ arm circumference taken halfway between the acromion and olecranon processes. Additionally, the participants’ arm was supported at heart level. More specifically, the middle portion of the cuff was aligned with the right atrium, at the midpoint of the sternum.

### R–R signal acquisition and heart rate variability processing

The R–R intervals were recorded at a frequency of 1000 Hz, providing an accuracy of 1 ms for each R–R interval. Recorded R–R intervals were first transferred to Polar Precision Performance Software and visually inspected for undesirable premature beats and noise. An R–R interval was interpreted as premature if it deviated from the previously quantified interval by >30%. No premature beats were observed in the complete set of R–R intervals obtained from each individual; therefore, there was no need for interpolation due to ectopy.

Power spectral analysis was computed after data detrending. Spectral decomposition of heart rate variability was conducted using an autoregressive approach. The autoregressive spectrum was calculated by fitting a 16th-order model to the R–R data [24]. The raw power was calculated by measuring the area under the peak of the power spectra density curve and corresponding bandwidths interpreted as follows: high frequency (HF) component (0.15–0.4 Hz) indicative of cardiovagal modulation; low frequency (LF) component (0.04–0.15 Hz) reflecting a combination of sympathetic and vagal cardiac modulation. The ratio of LF/HF (low to high frequency power ratio) was then calculated and used as an index of sympathovagal balance [25]. All data acquisition and post-acquisition analyses were carried out using Kubios HRV Analysis Software 2.0 for Windows (The Biomedical Signal Analysis Group, Department of Applied Physics, University of Kuopio, Finland) and in accordance with standards put forth by the Task Force of the European Society of Cardiology and North American Society of Pacing and Electrophysiology [26].

### Statistical analysis

All data are reported as mean ± S.D. Before comparing both conditions (50 ml compared with 500 ml), data were tested for normality and homoscedasticity with the Kolmogorov–Smirnov and Levene's tests respectively. Data were analysed by an independent researcher in our laboratory who was blinded to treatment allocation. Based on the preliminary findings of one previous report [27], if the true difference in mean arterial pressure (MAP) post-drinking 500 ml of water ± within group S.D. is 8.7±6.4 mmHg for women and 1.9±4.9 mmHg for men, a total of 31 participants (16 men and 15 women) was estimated to have more than 80% power of correctly rejecting the null hypothesis (minimum of 13 participants in each group to yield power of 80%). Independent Student's *t* tests were used to determine sex differences in descriptive characteristics. A three-way ANOVA [sex (women compared with men) by condition (50 ml compared with 500 ml of water) by time (pre- compared with post-water ingestion)] with repeated measures was conducted on all dependent variables to determine the effects of water ingestion on resting haemodynamics.

We obtained significant haemodynamic differences between men and women at baseline. To control for these differences, the percent change from pre- to post-ingestion time point was calculated for each volume of water on an individual basis. Subsequently, potential sex differences in the haemodynamic response to each volume of water were re-evaluated with one-way MANOVAs using a set of four dependent variables (systolic BP, diastolic BP, MAP and heart rate). Additionally, as the magnitude of the osmopressor response depends on water dosage [2], and men were heavier than women, we also computed these analyses using body mass as a covariate (MANCOVAs). Adjustment for multiple comparisons was made with the Bonferroni's correction. LF and HF power were transformed to their natural logarithm (ln) for statistical analysis because of their skewed distribution. All statistical calculations were computed using SPSS version 21.0 and a significance level of *P*<0.05 was used.

## RESULTS

Table 1 summarizes the participant characteristics. Compared with men, women had lower height, body mass and BMI, whereas age was similar between sexes. The between-sex comparisons, before and after water ingestion, are shown in Table 2. We obtained a sex main effect for systolic BP (*F*=13.4, *P*<0.05), diastolic BP (*F* =9.5, *P*<0.05), MAP (*F*=7.7, *P*<0.05) and heart rate (*F*=4.2, *P*<0.05); thus indicating that overall arterial pressure values were lower, whereas resting heart rate was higher in women than men. There were significant condition-by-time interactions for diastolic BP (*F*=9.5, *P*<0.05), MAP (*F*=7.2, *P*<0.05) and heart rate (*F*=10.2, *P*<0.05). Post hoc analyses revealed no significant differences in resting haemodynamics before the ingestion of either volume of water. Nevertheless, drinking 500 ml of water elicited an increase in diastolic BP and MAP (*P*<0.05); however, this was not the case for the 50 ml condition. Similarly, there was a reduction in heart rate post-500 ml of water (*P*<0.05) but not after drinking 50 ml of water. Finally, repeated measures ANOVA indicated no other significant interactions involving sex, condition or time resulting from water ingestion.

**Table 1:** Descriptive characteristics of the participants Values are mean ± S.D. *Sex differences (*P*<0.05).

Variables	Women (*n*=15)	Men (*n*=16)
Age (years)	24.8±6.1	24.2±7.1
Height (cm)*	166.5±7.4	175.7±6.5
Body mass (kg)*	59.3±7.6	74.4±6.7
Body mass index (kg/m^2^)*	21.3±1.5	24.1±1.8

**Table 2:** Haemodynamic variables obtained at rest in women and men before and after the ingestion of each volume of water Values are mean ± S.D. Abbreviation: DBP, diastolic blood pressure; MAP, mean arterial pressure; SBP, systolic blood pressure; *Sex main effect (*P*<0.05); ^†^condition-by-time interaction (*P*<0.05).

Variables	50 ml		500 ml	
	Pre-water	Post-water	Pre-water	Post-water
SBP (mmHg)*				
Women	106.5±6.6	109.1±9.3	106.7±8.9	114.3±10.5
Men	117.3±8.5	117.4±8.5	119.2±8.9	120.9±11.3
DBP (mmHg)*^†^				
Women	67.1±6.9	69.91±5.9	65.3±8.0	73.3±8.3
Men	72.1±7.3	71.5±5.5	74.5±6.1	77.6±6.3
MAP (mmHg)*^†^				
Women	81.5±6.9	84.7±7.9	80.5±8.2	88.2±8.8
Men	88.4±8.1	87.9±6.1	90.6±6.5	93.5±7.7
Heart rate (bpm)*^†^				
Women	73.1±10.2	71.0±9.3	72.4±10.7	66.7±7.8
Men	59.3±6.5	60.4±5.9	59.1±8.9	56.4±6.8

**Figure 1:**
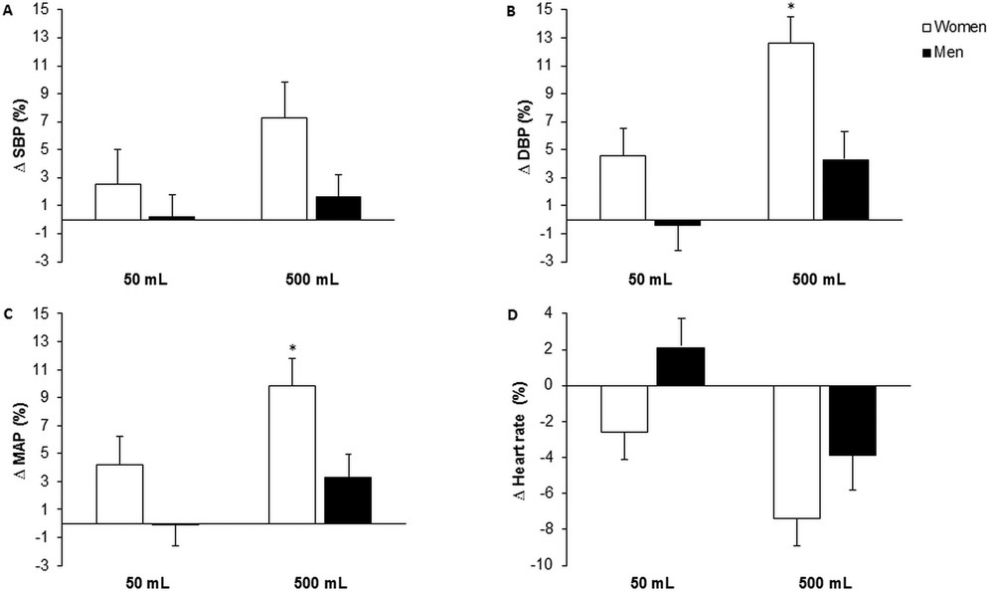
Haemodynamic response to water ingestion in men and women (50 ml compared with 500 ml) The bars indicate mean ± S.D. percent change (from pre- to post-ingestion time point) in (**A**) systolic blood pressure (SBP), (**B**) diastolic blood pressure (DBP), (**C**) mean arterial pressure (MAP) and (**D**) heart rate. *Sex differences (*P*<0.05).

We then calculated the percentage change in BP and heart rate from pre- to post-ingestion time point on an individual basis. These data were examined for sex differences using one-way MANOVAs. This allowed us to compare the haemodynamic response to each volume of water, between men and women, while controlling for sex differences in resting haemodynamics. Multivariate analysis indicated a significant sex main effect for resting haemodynamics after 500 ml of water (Wilks Lambda=0.6, *F*=4.2, *P*<0.05). Follow up univariate analyses on haemodynamic data demonstrated that, in comparison with men, women responded to 500 ml of water with a greater magnitude of change in diastolic BP (*F*=9.4, *P*<0.05) and MAP (*F*=7.8, *P*<0.05) (Figure 1). Conversely, the percent change in systolic BP and heart rate was not different between sexes in the 500 ml condition. No sex differences were seen in the percent change of BP or heart rate post-drinking 50 ml of water. The results from MANCOVAs, controlling for body mass differences between sexes, provided similar results (multivariate analysis–Wilks Lambda =0.6, *F* =3.5, *P*<0.05; univariate analyses–diastolic BP: *F* =12.3; MAP: *F*=12.9, *P*<0.05).

The effects of water ingestion on the power spectrum of heart rate variability are shown in Table 3. As can be seen, we obtained no sex differences for resting measures of heart rate variability at either condition or time point. Both the raw power of LF and the LF/HF ratio were similar between conditions at baseline and did not respond to either volume of ingested water. Conversely, there was a significant condition-by-time interaction for HF raw power (*F*=12.5, *P*<0.05); thus indicating that the ingestion of 500 ml of water resulted in an overall increase in HF power compared with that seen post-50 ml of water. Importantly, the proportion of this response was not different between men and women.

**Table 3:** Spectral components of heart rate variability obtained at rest in women and men before and after the ingestion of each volume of water Values are mean ± S.D. Low frequency power (LF) and high frequency power (HF) are the natural logarithm (ln); low to high frequency power ratio (LF/HF). ^†^Condition-by-time interaction (*P*<0.05).

	50 ml		500 ml	
Variables	Pre-water	Post-water	Pre-water	Post-water
LF (ln ms^2^)				
Women	6.5±0.8	7.1±0.8	6.5±0.8	7.1±0.6
Men	6.8±0.9	7.3±0.7	6.9±0.6	7.3±0.5
HF (ln ms^2^)^†^				
Women	6.8±1.1	6.9±1.2	6.9±1.1	7.6±1.0
Men	7.1±1.2	7.0±1.0	7.0±0.9	7.4±1.0
LF/HF				
Women	1.6±3.0	2.1±2.5	1.6±1.7	1.2±1.2
Men	1.3±1.3	1.9±1.6	1.4±1.4	1.6±1.7

## DISCUSSION

In support of our hypothesis, we observed that the magnitude of the osmopressor response resulting from the intake of a single water bolus of 500 ml follows a sexually dimorphic pattern. Specifically, it was demonstrated that the overall haemodynamic response to water ingestion is more pronounced in women than in men. Moreover, the findings from the present study suggest that sex differences post-water ingestion are largely limited to its impact on diastolic BP and MAP. Additionally, it was shown that these results were not related to different body mass values between sexes (i.e. not dependent on the volume of water ingested per kilogram of body mass). Despite this, both men and women demonstrated a similar increase in cardiovagal drive post-drinking 500 ml of water.

Past research indicates that the haemodynamic response to water ingestion is independent of changes in plasma renin activity, vasopressin or blood volume [2] and there is compelling evidence that it is secondary to the activation of the transient receptor potential cation channel family (TRPV4 osmolarity-sensitive Ca^2+^ channels) within the hepatic spinal afferents, dorsal root ganglia and spinal cord [28]. More recently, it was also shown that the osmopressor effect occurs simultaneously with the up-regulation of aquaporin-1 tyrosine phosphorylation on red blood cells [5]. This is important because the sympathetic nervous system is a major target of osmoregulatory neural pathways. The increase in venous plasma noradrenaline levels, MSNA, as well as sweating after water ingestion provides evidence of hypoosmolality-stimulated sympathetic activation [29]. We found that drinking 500 ml of water elicited a greater magnitude of change in diastolic BP and MAP in women compared with men; thus indicating a likely role for sex in the regulation of post-water ingestion afferent receptor stimulation, reflex mediated control of peripheral sympathetic outflow and regulation of neurotransmitter release from peripheral sympathetic nerve terminals and the adrenal medulla.

In the present study, women had lower BP, but higher heart rate values than men at resting conditions. Our findings agree with those of previous investigations because haemodynamic measurements have consistently found that men have higher BP and lower heart rate than premenopausal, age-matched women [30,31]. Numerous studies in humans and animals have examined sex differences in cardiovascular autonomic regulation. Experimental data has provided conflicting results on whether sex hormones can increase or decrease sensitivity of blood vessels for α-adrenergic agonist-induced constriction. Although some investigations have found men to be more sensitive to the vasoconstricting effects of phenylephrine [17,32–34], other reported the exact opposite [19,35,36]. However, it is important to point out that the direction of sex differences in α-adrenergic vasoreactivity varies depending on the part of the body where the effect is explored. For example, it has been shown that phenylephrine elicits greater isometric tension in male rat aortic strips and a more pronounced reduction in blood flow through the arteries of the upper extremities in men [17,32–34]. Conversely, other reports described that oestrogens increase the affinity of α-adrenergic receptors in mesenteric arteries and that this phenomenon underlies heightened sensitivity to catecholamine-induced vascular contraction in female rats [35,36]. In humans, the available data indicate that splanchnic and cutaneous blood flow is reduced in women compared with men [18,37,38]. Therefore, even though the mechanism(s) by which oestrogen exerts differential effects on α-adrenergic affinity within disparate vascular networks is unknown, the existent data strongly indicate that female resistance vessels (e.g. splanchnic and cutaneous circulation) are particularly reactive to pressor agents. In contrast, female conduit vessels (e.g. aorta and brachial artery) are less responsive to α-adrenergic stimulation compared with that seen in male arteries. Given that the ingestion of 500 ml of water has been shown to be particularly effective in decreasing skin blood flow, in concert with a reciprocal increase in vascular peripheral resistance [39], we speculate that sex differences in the osmopressor response might partially originate from augmented cutaneous vasoconstriction in women compared with men. As importantly, the afferent neurons that innervate hepatic portal vessels and sense osmolality changes are enriched in the lower thoracic dorsal root ganglia [28]; lying close to the spinal cells that originate presynaptic input to the sympathetic celiac and superior mesenteric ganglia. Accordingly, although bearing in mind that female splanchnic circulation is more responsive to variations in catecholaminergic constrictor tone, the osmopressor response may also be more pronounced in women because water ingestion activates portal osmosensors that ultimately synapse with cell bodies of preganglionic fibres responsible for mesenteric vasoconstriction [28,36].

In healthy young individuals, there is general agreement that increased cardiovagal drive counteracts the effects of the elevated sympathetic tone post-water drinking; thus largely attenuating [6,13] or even dissipating [3,4,11] the change in resting BP. We observed a similar reduction in resting heart rate between men and women post-drinking 500 ml of water (both in absolute and relative terms). Moreover, as negative chronotropism was accompanied by heightened HF power spectral modulation in both sexes, our findings likely suggest that it was of vagal origin. No between-sex differences were seen for the magnitude of increase in the raw power of HF post-drinking 500 ml of water. Thus, by showing that men and women respond to the vasoconstrictive effects of water ingestion with similar compensatory changes in cardiovagal drive; our findings further extend those of previous studies. Nevertheless, when using the percent change from baseline as an outcome, we found that the water pressor effect was significantly greater in female than in male participants. Additionally, these results were not related to different body mass values between sexes. Thus, in women, circulatory adjustments post-water drinking may not fully compensate for the effect of increased vascular resistance on resting BP. In agreement with this concept, previous reports have shown that the reflex bradycardia that occurs secondarily to increases in arterial BP is diminished in women compared with men [15,40]. The most likely cause of sexual dimorphism in the bradycardic response to elevations in BP is a reduced gain of cardiovagal baroreflex in women [15,41]. Finally, from a physiological standpoint, it has been suggested that an attenuated cardiovagal baroreflex response might result from lower levels of female carotid artery distensibility [42,43]; thus resulting in a smaller mechanical transduction of arterial pressure into barosensory stretch.

There is general agreement that cardiovascular autonomic function is sensitive to the hormonal changes occurring within different phases of the menstrual cycle [16,44,45]. Yet, previous research has neglected to control for its effects on the osmopressor-response to water ingestion. It is possible that the lack of control for the effects of the menstrual cycle phase on the physiological responses to water ingestion may have contributed to the discrepancies reported in the existent literature on healthy young adults. In the early follicular phase, there is greater vasoconstriction mediated by the α_2_-adrenoceptors [16]; which are known to be more prominent than α_1_-adrenoceptors in human resistance arteries [46]. Conversely, resting MSNA and circulating noradrenaline levels are substantially higher during the mid-luteal phase (when both oestrogen and progesterone are markedly elevated) [15]. Based on these data, it is difficult to predict which (if any) menstrual phase is characterized by a more pronounced haemodynamic response to water ingestion. Future research is warranted to examine the impact of the menstrual cycle on the osmopressor response to water ingestion in healthy, young women.

We conclude that, in the early follicular phase of their menstrual cycle, women exhibit a greater magnitude of increase in resting BP post-water ingestion compared with men of similar age. Accordingly, our study provides direct evidence of sexual dimorphism in the haemodynamic response to water intake in young healthy adults.

### Practical implications

Ingestion of water is proven therapeutic to relieve debilitating hypotension episodes, such as those resulting from prolonged orthostatism, post-exercise passive recovery, blood donation and ingesting a meal [6,14,47–50]. Since women have greater incidence of orthostatic intolerance than men, water drinking may provide an effective and inexpensive strategy for delaying the onset of presyncopal and syncopal events [18].

### Limitations

The present study has at least six important limitations. (1) BP was measured using a non-continuous cuff-based method and this approach has several disadvantages when compared with beat-to-beat non-invasive technology (e.g. continuous BP recoding is not possible, short-term changes in BP cannot be detected and cuff inflation may disturb the person being tested; thus affecting the quality of the measurements) [51]. (2) We did not measure baroreflex gain, total peripheral resistance, skin or splanchnic blood flow at either time point. Therefore, based on previous research, we can only speculate about the origin of sex differences found after drinking 500 ml of water. (3) Our experimental design only included women in the early follicular phase. Accordingly, the present results may not be sustained during alternative phases of the menstrual cycle (e.g. luteal phase). (4) We only tested healthy young adults. Since the physiological responses to water ingestion are known to vary as a function of the aging process and the degree of autonomic failure [2,12], generalizations based on our findings are not possible. (5) Physical condition of the participants was not taken into consideration in the present study. Although it has been suggested that different levels of physical condition may affect cardiovascular responses to stress [52], this has not been demonstrated in studies similar to this one examining the haemodynamic response to a single water bolus of 500 ml of water. As importantly, past research also indicates that the lower level of cardiovagal baroreflex gain in women is not associated with between-sex differences in aerobic fitness [15]. Therefore, we do not believe that our data were substantially influenced by different levels of physical conditioning in participants from either group. (6) There were significant differences between groups for BMI. Even though men exhibited higher BMI than women, the clinical relevance of this is questionable because the mean values of both groups was between normal values (i.e. 18.5 and 24.9 kg/m^2^) [-5].

## Abbreviations

BMI: body mass index
BP: blood pressure
HF: raw high frequency power
LF: raw low frequency power
LF/HF: low to high frequency power ratio
ln: logarithm
MANCOVA: multivariate analysis of covariance
MANOVA: multivariate analysis of variance
MAP: mean arterial pressure
MSNA: muscle sympathetic nerve activity
SBP: systolic blood pressure
TRPV4: transient receptor potential cation channel subfamily vanilloid 4
